# Decreased TUSC3 Promotes Pancreatic Cancer Proliferation, Invasion and Metastasis

**DOI:** 10.1371/journal.pone.0149028

**Published:** 2016-02-12

**Authors:** Xiaoqiang Fan, Xiu Zhang, Jie Shen, Haibin Zhao, Xuetao Yu, Yong’an Chen, Zhuonan Zhuang, Xiaolong Deng, Hua Feng, Yunfei Wang, Long Peng

**Affiliations:** 1 Department of Oncology, Jimin Hospital, Shanghai, China; 2 Department of Pathology, Taihu Hospital, Wuxi, Jiangsu Province, China; 3 Department of General Surgery, Shandong University Affiliated Qilu Hospital, Ji’nan, Shandong Province, China; Wayne State University School of Medicine, UNITED STATES

## Abstract

Pancreatic cancer is an aggressive disease with dismal prognosis. It is of paramount importance to understand the underlying etiological mechanisms and identify novel, consistent, and easy-to-apply prognostic factors for precision therapy. TUSC3 (tumor suppressor candidate 3) was identified as a potential tumor suppressor gene and previous study showed TUSC3 is decreased in pancreatic cancer at mRNA level, but its putative tumor suppressor function remains to be verified. In this study, TUSC3 expression was found to be suppressed both at mRNA and protein levels in cell line models as well as in clinical samples; decreased TUSC3 expression was associated with higher pathological TNM staging and poorer outcome. In three pairs of cell lines with different NF-κB activity, TUSC3 expression was found to be reversely correlated with NF-κB activity. TUSC3-silenced pancreatic cancer cell line exhibited enhanced potential of proliferation, migration and invasion. In an orthotopic implanted mice model, TUSC3 silenced cells exhibited more aggressive phenotype with more liver metastasis. In conclusion, the current study shows that decreased immunological TUSC3 staining is a factor prognostic of poor survival in pancreatic cancer patients and decreased TUSC3 promotes pancreatic cancer cell proliferation, invasion and metastasis. The reverse correlation between NF-κB activity and TUSC3 expression may suggest a novel regulation pattern for this molecule.

## Introduction

Pancreatic cancer is an aggressive disease with dismal prognosis. It is the ninth most common cancer in the world but ranks fourth of cancer related deaths [1], with approximately 227,000 people dying from this disease each year [2]. Surgical resection remains the only option for cure. Nevertheless, majority of the patients are discovered at later stage for lack of specific symptoms, losing the chance of curative resection. Even those who undergo resection with curative intention will generally relapse within two years [3]. Conventional chemotherapeutic agents and the novel molecular target drugs have modest effects on disease course [4–6], and only slightly improve disease free survival in an adjuvant therapy setting [3, 7]. Stratification of patients according to recurrence risk will help maximize the personalization of intervention and enhance therapeutic efficacy with minimum side effects. In recent years, many pathological factors have been established for predicting prognosis including tumor size, tumor grade, vascular invasion, lymph node metastasis and perineural invasion [8]. Systemic inflammatory index and circulating tumor markers such as CA199 were also explored for their prognostic value [9–12]. There are also some molecular markers that are known to be predictive of treatment efficacy and long-term outcome such as Kras [13], EGFR, and Her2/neu et al [14]. However, they are still insufficient for patients consulting and individualized therapy. It is of paramount importance to understand the mechanisms that contribute to the progression of the disease and identify novel, consistent, and easy-to-apply prognostic factors for precision therapy.

TUSC3 (tumor suppressor candidate 3), was identified as a potential tumor suppressor gene previously. It is located on chromosomal band 8p22, which is frequently deleted in several tumor types including prostate [15] and pancreatic cancer [16–18]. Therefore, TUSC3 is supposed to be a tumor suppressor gene. It is a homologue of the yeast Ost3p subunit of the oligosaccharyltransferase (OST) complex, an integral membrane protein complex that catalyzes N-linked glycosylation of proteins in the endoplasmic reticulum (ER) [19, 20]. Later on, TUSC3 was also found to be involved in Magnesium transport and oxidoreductase activity [21]. As N-glycosylation is a ubiquitous posttranslational modification of eukaryotic proteins by modulating protein folding, protecting them from degradation, and regulating their function as well as their immunogenicity, decreased TUSC3 is supposed to be implicated in cancer pathogenesis via glycosylation [22]. Krainer and his colleagues tested TUSC3 expression in 143 prostate patients using a tissue microarray, and found that 13.3% of tissue samples demonstrated starkly reduced protein expression of TUSC3, but follow-up of the cohort failed to show TUSC3 influence on progression-free or overall survival [23]. In ovarian, TUSC3 was also found to be significantly down-regulated in higher-grade ovarian cancer specimens [24]. Further study showed its low expression results from TUSC3 promoter hypermethylation and the methylation status of the TUSC3 promoter has a significant and independent influence on progression free and overall survival for ovarian cancer patients [25]. As early as in 2005, homozygous and heterozygous deletion of TUSC3 gene was discovered in pancreatic cancer cell lines and specimens with genomic array-based studies [16]. Nevertheless, its putative tumor suppressor function remains to be characterized in this disease. What also remains to be clarified is whether its expression is associated with clinicopathological staging and whether it carries a prognostic value for this cancer entity.

Our aim is to explore whether TUSC3 expression is associated with pancreatic cancer clinicopathological features and represents a prognostic factor for this cancerous disease and further characterize the function of the molecule in the pathogenesis of the disease using cell line models and a mouse model.

## Materials & Methods

### Patients & Samples

Paraffin-embedded specimens from 117 patients with pancreatic cancer (PC) who underwent surgical resection between January 2002 and December 2012 at our hospital were retrieved. Samples from 69 men and 48 women with a mean age of 60.2 years (range, 42 to 71 years; median age, 61 years) were found. The median follow-up was 19 months (range, 1 to 69 months.) The study conformed to the ethical guidelines of the 1975 Declaration of Helsinki and was approved by the committee of ethics of Jimin Hospital, Shanghai, China. All patients signed a written informed consent of this study.

### Immunohistochemistry

5μm thick tissue sections were deparaffinized by heating at 60°C and subsequently rehydrated in xylene and graded alcohols. Antigen retrieval was performed with DEPP-9 epitope retrieval solution, followed by treatment with 0.3% H_2_O_2_ in PBS (pH7.4) to quench endogenous peroxidase activity. After blocking with 10% secondary antibody host serum for 10 minutes, the sections were incubated in primary antibody (rabbit polyclonal to TUSC3,dilution 1:300, ab65213, Abcam, Cambridge, UK) for 1 hour at room temperature. Primary antibody dilutions were made in 10% secondary antibody host serum. The sections, after 2×PBS washes, were incubated in respective biotinylated secondary antibodies [biotinylated anti-rabbit IgG (BA-10000), Vector Laboratories, Burlingame, CA, USA], diluted 1:200 in 10% serum for 30 minutes at room temperature, followed by 45 minute incubation in StreptABComplex/HRP (K0377 Dako, Glostrup, Denmark). The sections were again washed twice with PBS and incubated in Dako Liquid DAB+ Substrate-Chromogen System (K3468) until the development of brown color. This was followed by counterstaining with Meyer’s hematoxylin, dehydration, and mounting using Eukitt medium.

Each slide was stained in serial sections and examined by 2 pathologists. The intensity of the staining was scored as follows: 0, negative; 1+, weak staining; 2+, moderate staining; 3+, strong staining. For semi-quantitative analysis, H-score [26] was used in this study. Briefly, more than 500 tumor cells were counted in each case, and the H-score was subsequently generated by adding the values of percentages of strong staining multiplied by 3 plus percentage of moderate staining multiplied by 2 plus percentage of weak staining multiplied by 1., giving a possible range of 0–300. H-score more than 100 was noted as high expression. We also examined TUSC3 expression in the lymph node (LN) metastasis in 24 cases and compared the TUSC3 expression differences between the primary lesion and lymph node metastasis. Ki 67 was scored in the manner of percentage, i.e. labeling index, regardless of immunostaining intensity.

### Cell culture conditions

Fifteen human PC cell lines and one immortalized non-tumorigenic pancreatic epithelial cell line (HPNE) were chosen for this study. All the cell lines were obtained as gifts from Dr Paul J. Chiao’s lab in MD Anderson, Texas University. AsPC-1, AsPC-1 mu [27], MDA pan28, MDA p28 mu [28], BxPC-3, Colo357, L3.6pl, MIAPaCa-2, PANC-1, PTAC43, PTAC50, PTAC53, PTAC66, and Capan-1 Cells were grown as a monolayer cell culture in DMEM containing 4.5 mg/ml D-glucose and L-glutamine supplemented with 10% fetal bovine serum. HPNE cells were grown in Medium D with a mixture of M3 medium and DMEM containing one volume of M3 Base F culture medium (InCell Corp., San Antonio, USA), three volumes of glucose-free DMEM, 10% FBS, 5.5 mM glucose, 10 ng/ml EGF, and 50 mg/ml gentamycin. The 293T cell line was grown in complete DMEM. HPNE/Kras/p16shRNA and HPDE/Kras/Her2/p16shRNA/smad4shRNA were pancreatic cancer cell lines (a gift from Dr Paul J. Chiao’s lab, The University of Texas MD Anderson Cancer Center) by transfection of relevant plasmid into HPNE and HPDE cell respectively[29]. HPDE/Kras/Her2/p16shRNA/smad4shRNA cells were grown in keratinocyte serum-free medium.

### Cell proliferation and Clonogenic Assay

For the cell growth curve assay, cells were plated in 24-well plates (1×10^4^ cells/well) in triplicate in growth medium. Cells were counted at days 1, 3, and 5. Results were expressed as the means ±standard error of the mean from three independent experiments. To test the clonogenecity of the TUSC3 silenced PC cells, trypan blue exclusion tested viable TUSC3 knockdown cell lines and the scramble control cells were plated (100/well) in a six-well plate and then incubated for about 10–12 days at 37°C in a 5% CO_2_/5% O_2_/90% N_2_ incubator. The colonies were stained with 2% crystal violet. A representative field for each experiment was photographed at 100 ×magnification. At least three independent assays were performed in triplicate. Data were shown as the mean value of the number of colonies/per field ± the standard error of the mean from three independent experiments.

### Real-time PCR

Total RNA was extracted from the cells by applying TRIzol according to the manufacturer’s protocol (Invitrogen). RNA quality and quantity were measured by an ND-2000 spectrophotometer (Nanodrop). cDNA was synthesized using the PrimeScript RT Master Mix (Bio-Rad). cDNA (20 ng) was subjected to real-time PCR performed with SYBR reagents using the IQ5 PCR system (Bio-Rad, Hercules, CA). β-actin was used as the internal control gene. Specific primers were synthesized by Sigma-Aldrich and the sequences were described as follows: hTUSC3: forward primer: 1275-AGACGGATAATTTGCCTAGTGGG-1297, reverse primer: 1417-AGTGCCATGGTCCAAATCACA-1397. The plot compares the relative expression level of mRNA in several pancreatic tumor cell lines compared to the immortalized non-tumorigenic pancreatic epithelial cell HPNE. Each experiment was performed three times and each time with three replicates.

### Lentiviral transduction

Knockdown of TUSC3 in pancreatic cancer cell line Colo357 was performed by lentiviral delivery using Plko.1 vector containing TUSC3 shRNA (SKU: SR305331, OriGene Technologies, CO. USA), and HEK293T packaging cell line. Transduced cells were selected and maintained in medium containing puromycin (3ug/ml).

### Matrigel assay

50,000 cells were seeded in triplicates onto a 24-well plate with modified Boyden chambers (BD Biosciences, San Jose, CA, USA). The lower chamber contained 10% FCS/culture medium as chemoattractant. Cells were incubated for 18 hours, stained with crystal violet and counted under a microscope.

### Immunoblotting

Cells were lysed with RIPA buffer supplemented with complete Protease Inhibitor Cocktail Tablets (Roche Diagnostics, Mannheim, Germany) and PhosSTOP Phosphatase Inhibitor Cocktail Tablets (Roche Diagnostics). After incubation for 10 minutes on ice, cell lysates were cleared by centrifugation at 15,000 rpm for 10 minutes at 4°C, and protein concentration was determined by Bradford absorbance assay (Sigma Aldrich). Equal amounts of protein lysates(40μg) were separated by SDS-PAGE, blotted on PVDF membranes (GE Healthcare, Chalfont St. Giles, UK), incubated with the appropriate primary antibody and horseradish peroxidase (HRP)-conjugated secondary antibodies and detected with enhanced chemiluminescence detection system (Pierce ECL Western Blotting Substrate, Thermo Scientific, Rockford, IL, USA). Following antibodies and dilutions were used: β-actin (1:500, sc-1616, Santa Cruz Biotechnology, Santa Cruz, CA), TUSC3 (1:300, ab65213, Abcam, Cambridge, UK).

### Animals and Orthotopic Implantation of Pancreatic Tumor Cells

Balb/C nude mice were purchased from the Animal Production Area of the National Cancer Institute Frederick Cancer Research and Development Center (Frederick, MD). The mice were housed under specific pathogen-free conditions in facilities approved by the American Association for Accreditation of Laboratory Animal Care and in accordance with current regulations and standards of the U.S. Department of Agriculture, U.S. Department of Health and Human Services, and the National Institutes of Health. The mice were used in accordance with institutional guidelines when they were 8 to 12 weeks old.

To produce pancreatic tumors, TUSC3 silenced PC cells were harvested from subconfluent cultures by a brief exposure to 0.25% trypsin and 0.02% EDTA. Trypsinization was stopped with medium containing 10% fetal bovine serum, and the cells were washed once in serum-free medium and resuspended in Hanks’ balanced salt solution (HBSS). Only suspensions consisting of single cells with greater than 90% viability were used for the injections. One million cells suspended in 50μl of HBSS were injected into the pancreas of nude mice as described previously. The mice were killed after 5 weeks injection and necropsied. The size and weight of the liver metastases were recorded.

### Statistical analysis

All statistical computations were performed using the Graph Prism 5.0 software (San Diego, CA, USA). Cox regression was performed with SPSS version 19.0 (SPSS, Inc., Chicago, IL, USA). Comparison of means in normally distributed data was performed using Students’ t test otherwise the nonparametric Mann-Whitney U test was applied or as stated. P-values of equal or less than 0.05 were considered statistically significant. All bar graphs are depicted using means and standard deviations as error bars, unless stated otherwise.

## Results

### TUSC3 expression in clinical samples

**Fig 1:**
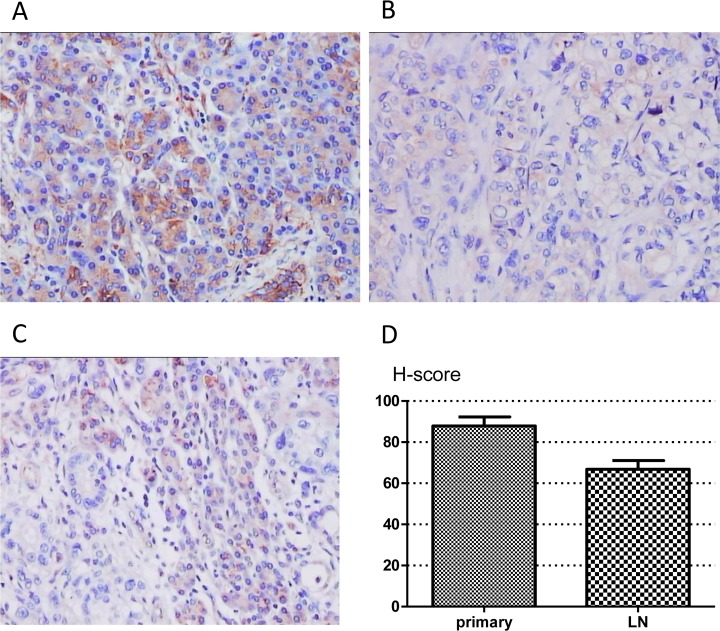
TUSC3 is decreased in pancreatic cancer samples. (A) TUSC3 expression in non-neoplastic pancreatic tissues (acinar, duct and islets). (B) TUSC3 expression in pancreatic cancer cells. (C) Low TUSC3 expression in pancreatic cancer cells compared to islets. (D) Comparison of TUSC3 staining in primary PC and LN metastasis(paired t-test, p<0.001, n = 58). Original magnification×200.

Immunohistochemically, TUSC3 is absent in pancreatic stroma cells but almost all pancreatic duct epithelial cells, acinar cells and islets showed immunoreactivity (Fig 1A and 1C). We evaluated the correlation between expression data and clinical parameters, including patient age, sex, tumor size, location, grade, primary tumor classification (pT), lymph node metastasis (pN), distant metastasis (pM). TUSC3 expression was observed to various degrees in pancreatic carcinoma cells in cytoplasm (Fig 1B and 1C). TUSC3 was detected in 38/117 (32.48%) pT specimens with H-score 83.42±2.882, in 14/58 (24.14%) pN specimens with H-score 66.81±4.265, and in 4/11 (36.36%) pM specimens with H-score 79.55±7.818. The expression of TUSC3 is dramatically decreased in metastatic LNs compared with the paired primary tumor samples (H-score: p<0.001) (Fig 1D).

### Decreased TUSC3 expression is associated with higher pathological TNM staging and poor prognosis

Overall, 69 male and 48 female patients with PC were included in this study cohort. The mean age at diagnosis was 64.6±10.3 years. Median survival was 19 months (range: 3–78 months). The tumor stage is defined as T_1_N_0_M_0_ in 8 patients (6.84%), T_2_N_0_M_0_ in 20 patients (17.09%), T_3_N_0_M_0_ in 24 patients (20.51%), T_1_N_1_M_0_ in 9 patients (7.69%), T_2_N_1_M_0_ in 18 patients (15.38%), T_3_N_1_M_0_ in 21 patients (17.95%), T_4_N_0_M_0_ in 2 patients (1.71%), T_4_N_1_M_0_ in 4 patients (3.42%), T_2-3_N_0_M_1_ in 5 patients (4.27%) and T_1-4_N_1_M_1_ in 6 patients (5.13%). 101 patients received chemotherapy, including 75 patients who received adjuvant chemotherapy, and 26 patients who received palliative chemotherapy. For 11 patients with liver metastasis discovered during operation, all viewable metastatic lesions were successfully removed.

The relationship between TUSC3 expression level and clinicopathological characteristics of patients with PC is shown in Table 1. Low TUSC3 expression was associated with larger tumor size (p = 0.039), higher serum CA199 level (p = 0.034), more lymphatic permeation (p = 0.031), and higher Ki-67 labeling index (p<0.001).

**Table 1:** TUSC3 expression is associated with clinical pathological staging and Ki67 index.

	TUSC3 expression		P value
	Low (n = 79)	High(n = 38)	
Age	59.6±0.8^a^	61.0±1.4^a^	0.33
Sex(male/female)	45/34	24/14	0.523
Tumor size>3cm	55(55/79)	19(50%)	0.039^b^
Differentiation (well+moderate/poor)	39/40	28/11	0.021^b^
Serum CA199 level>300IU/ml	59(59/79)	21(21/38)	0.034^b^
Clinicopathological features (%)			
Lymphatic permeation	50(50/79)	16(16/38)	0.031^b^
Vascular invasion	49(49/79)	21(21/38)	0.485
Perineural invasion	43(43/79)	18(18/38)	0.474
Liver metastasis	9(9/79)	2(2/38)	0.287
Ki-67 labeling index	51.70±8.123^a^	32.68±6.315^a^	<0.001^b^

a mean±SD

b p<0.05

**Fig 2:**
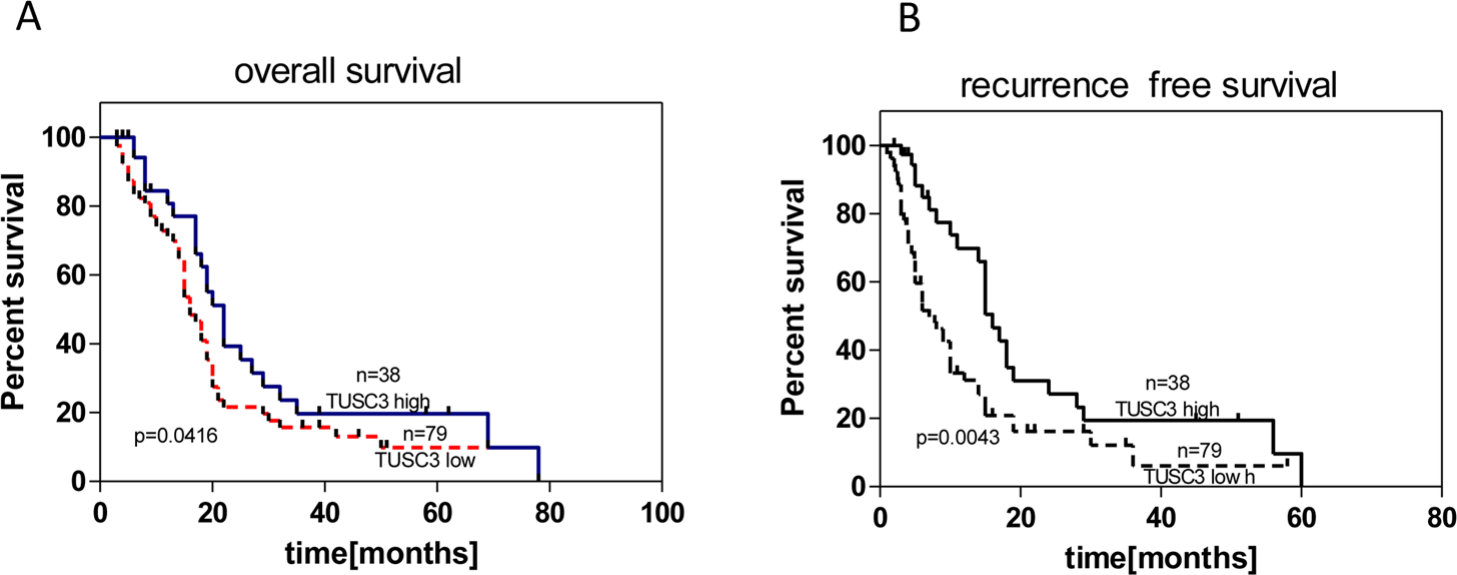
**Immunohistochemical staining of TUSC3 is associated with overall survival (A) and recurrence free survival (B) post curative resection of pancreatic cancer**.

To investigate whether TUSC3 protein expression level and other clinicopathological factors are associated with clinical outcome of PC patients, univariate and multivariate Cox proportional hazard models for CSS (Cancer Specific Survival) and RFS (Recurrence Free Survival) were calculated. Among the 117 patients, death occurred in 62 of 79 (78.5%) patients with low TUSC3 protein level and in 22 of 38 (57.9%) patients with high TUSC3 protein expression level (P<0.021). Fig 2A shows the Kaplan-Meier curves for CSS and reveals that low TUSC3 level is a factor predictive of poor prognosis in PC patients (p = 0.0416, log-rank test). Among the 117 patients, recurrence occurred in 69 of 79 (87.3%) patients with low TUSC3 protein level and in 24 of 38 (63.2%) patients with high TUSC3 protein expression level (p<0.0001). Fig 2B shows the Kaplan-Meier curves for RFS and reveals that low TUSC3 level is a factor predictive of higher risk of recurrence in PC patients (p = 0.0043, log-rank test).

Univariate analysis identified high tumor stage (stage I+II vs III vs IV P<0.001), high serum CA199 level (CA199<300IU/ml vs CA199≥00IU/ml, p = 0.034), no administration of chemotherapy (chemotherapy vs no treatment, p = 0.025), and decreased TUSC3 expression level (p = 0.015) as poor prognostic factors for RFS in this study cohort. And these factors are also predictive of poor overall survival in this cohort. Gender, age at diagnosis and tumor grade were not significantly associated with clinical outcome (Table 2).

**Table 2:** Univariate and Multivariate Cox Proportional Hazard Analysis of Factors Affecting Recurrence-free and Overall Survival in Pancreatic Cancer.

Variable	Univariate analysis		Multivariate analysis	
	HR(95%CI)	P	HR(95%CI)	P
Recurrence-free survival				
Age at diagnosis	1.08(0.99–1.15)	0.221	1.02(0.87–1.23)	0.30
TNM staging	2.98(1.95–4.76)	<0.001	3.12(2.10–4.54)	<0.001^a^
Tumor grade	1.16(0.87–1.42)	0.31	1.22(0.98–1.34)	0.222
CA199	1.61(1.15–2.02)	0.034	1.76(1.23–2.45)	0.027 ^a^
Adjuvant chemotherapy	1.43(1.02–2.17)	0.025	1.88(1.10–2.18)	0.017 ^a^
TUSC3 expression level	1.84(1.18–2.87)	0.015	1.68(1.08–2.88)	0.020 ^a^
Overall survival				
Age at diagnosis	1.10(0.88–1.21)	0.231	1.07(0.88–1.13)	0.334
TNM staging	3.18(2.05–3.76)	0.001	3.13(2.08–4.44)	<0.001 ^a^
Tumor grade	1.25(0.97–1.52)	0.357	1.22(0.98–1.54)	0.528
CA199	1.66(1.15–2.42)	0.030	1.76(1.22–2.25)	0.019 ^a^
Adjuvant chemotherapy	1.53(1.02–2.25)	0.034	1.89(1.11–2.48)	0.047 ^a^
TUSC3 expression level	1.88(1.28–2.77)	0.024	1.75(1.03–2.89)	0.027 ^a^

^a^ statistically significant.

To determine the independent prognostic value of the TUSC3 for CSS and RFS, a multivariate analysis using a Cox proportional hazard model was performed. In the multivariate analysis that included age, tumor grade, tumor stage, administration of chemotherapy, serum CA199 level and TUSC3 expression level, we identified tumor stage, administration of chemotherapy, and low TUSC3 protein expression level as independent prognostic factors for CSS and RFS(TUSC3 expression for RFS, p = 0.020; TUSC3 expression for CSS, p = 0.027, Table 2).

### TUSC3 expression is decreased in pancreatic cancer cell lines mediated by NF-κB

**Fig 3:**
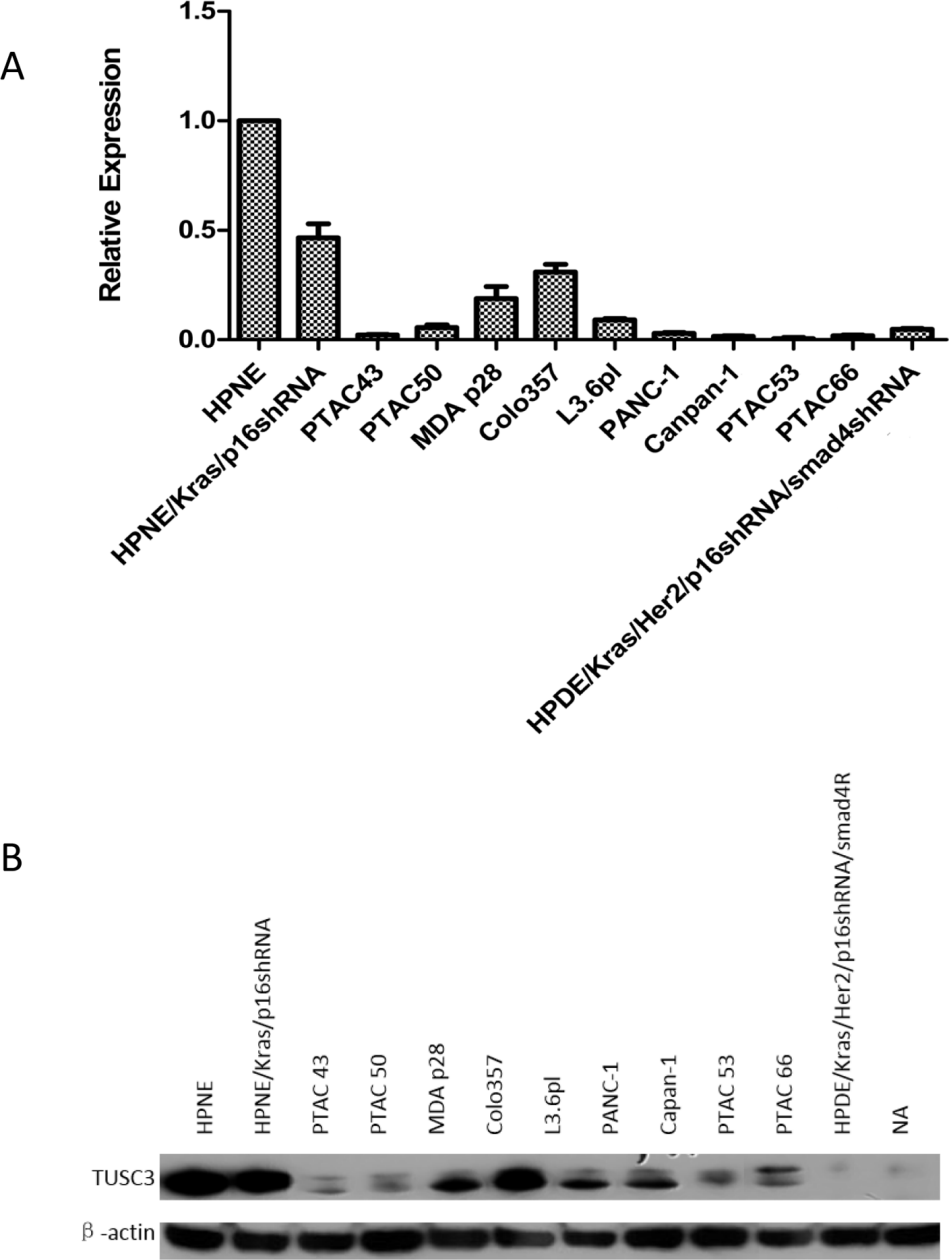
**TUSC3 is decreased in pancreatic cancer cell lines both at mRNA level (A) and protein level (B)**.

To characterize the *in vitro* function of TUSC3, we firstly examined the TUSC3 expression of different pancreatic cancer cell lines. Real-time PCR revealed TUSC3 was decreased at mRNA level in all the tested 11 pancreatic tumor cell lines (Fig 3A). Western Blotting revealed that it was also depressed at protein level in these cell lines (Fig 3B).

**Fig 4:**
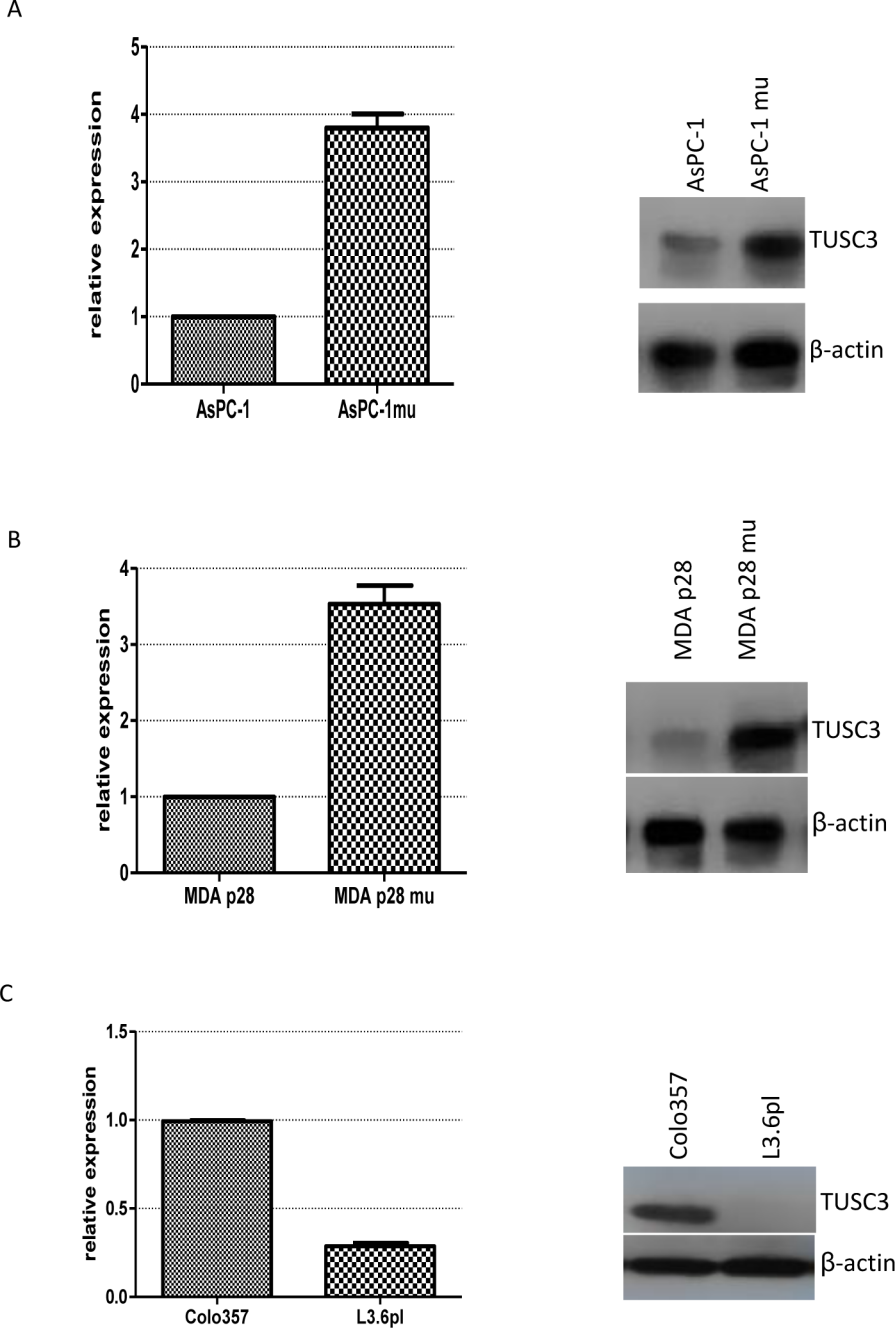
TUSC3 is regulated by NF-κB activity shown by three pairs of pancreatic cancer cell lines. (A) AsPC-1 and AsPC-1 mu with depressed NF-κB activity. (B) MDA p28 and MDA p28 mu with depressed NF-κB activity. (C) Colo357 and the daughter L3.6pl cell line with enhanced NF-κB activity. For each figure, left graph represents RT-PCR result, right graph represents Western blot for TUSC3.

To explore whether TUSC3 expression is correlated with NF-κB activity, we examined the TUSC3 expression both at RNA level and protein level in three pairs of pancreatic cancer cell lines with different NF-κB activity. AsPC-1 mu is a daughter cell fabricated by Paul J. Chiao et al by transfecting IκBαM (S32,36A) into the parental pancreatic cancer cell line AsPC-1, resulting in a tumor cell line with decreased NF-κB activity and decreased liver metastasis potential [27]. In this pair of cell model, it was demonstrated that TUSC3 expression of AsPC-1 mu is higher than that of AsPC-1 (Fig 4A), suggesting that TUSC3 is regulated by NF-κB activity. The same expression pattern is found in another pair of cell lines, MDA p28 and MDA p28 mu (Fig 4B). MDA p28 mu is a daughter cell line also produced in Dr Paul J. Chiao’s lab by transfecting IκBαM (S32, 36A) Iκ mu into MDA p28 cell line, resulting attenuated NF-κB activity and suppressed liver metastasis (data not published). TUSC3 expression is enhanced in MDA p28 mu compared with MDA p28. L3.6pl is a daughter cell line with much more potential of liver metastasis from consecutive injections of parental Colo357 into nude mice. It was demonstrated previously that L3.6pl exhibits higher NF-κB activity than Colo357 [30]. And interestingly, TUSC3 expression is higher in Colo357 than in L3.6pl both at mRNA and protein level (Fig 4C). All these three pairs of cell lines seemed to show the same pattern of TUSC3 expression in relevance to NF-κB activity which implies that TUSC3 is negatively correlated with NF-κB activity.

### Decreased TUSC3 promotes cell proliferation, migration and invasion

**Fig 5:**
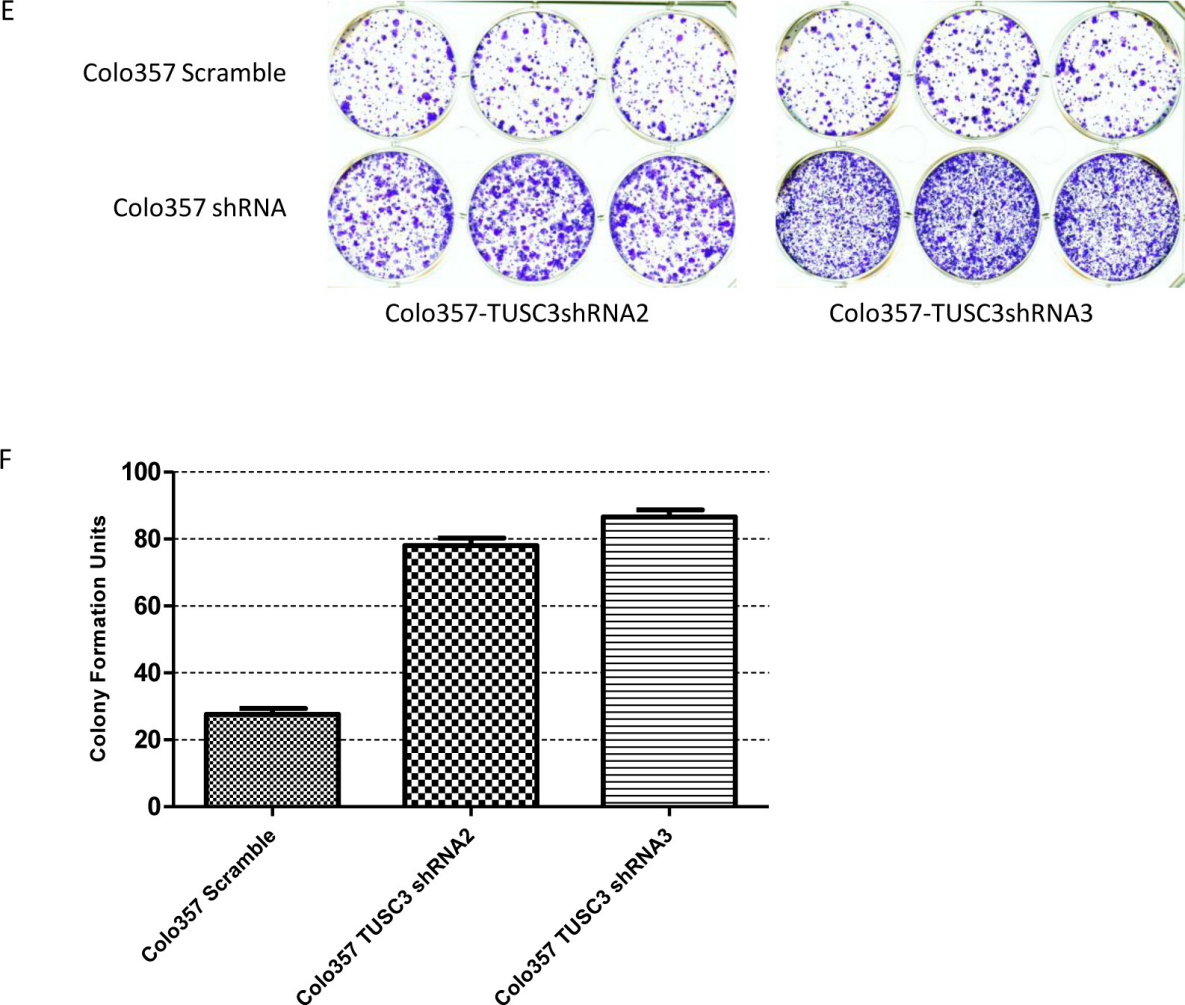
Decreased TUSC3 expression promotes tumor cell growth, migration and invasion. (A, B)TUSC3 is effectively knocked-down in Colo357 cell lines shown by (A) RT-PCR and (B) Western blot. (C, D) Proliferation is enhanced with TUSC3 knockdown, cell number counts are significantly different at 72 hours (Colo357 TUSC3 shRNA2 vs Colo357 Scramble p = 0.0086, Colo357 TUSC3 shRNA3 vs Colo357 Scramble p = 0.0011) (D). (E, F) Colony Formation is enhanced with TSUC3 knockdown (Colo357 TUSC3 shRNA2 vs Colo357 Scramble p<0.0001, Colo357 TUSC3 shRNA3 vs Colo357 Scramble p<0.0001). (G) Migration Test(Colo357 TUSC3 shRNA2 vs Colo357 Scramble p<0.0001, Colo357 TUSC3 shRNA3 vs Colo357 Scramble p<0.0001). (H, I) Invasion Test (Colo357 TUSC3 shRNA2 vs Colo357 Scramble p<0.0001, Colo357 TUSC3 shRNA3 vs Colo357 Scramble p<0.0001). (J) Wound healing test showed more rapid closure of the gap in TUSC3 silenced cells. All experiments were performed three times with representative figures shown as above.

To study the tumor suppressive function of TUSC3, shRNAs were designed to knockdown the expression of TUSC3 in *in vitro* pancreatic cancer model. As a parental cell line of a highly metastatic daughter cell with comparable NF-κB activities, Colo357 was chosen for the following experiment. Both mRNA and protein levels were detected to confirm the efficiency of silencing (Fig 5A and 5B). We investigated the effect of TUSC3 silencing on cell proliferation, colony formation, migration, and invasion conditions. Notably, After 72 hour of cultivation, the TUSC3 silenced cells gained a significant survival advantage in contrast to controls (Fig 5C and 5D). Colo357 TUSC3 shRNA2 vs Colo357 Scramble p = 0.0086, Colo357 TUSC3 shRNA3 vs Colo357 Scramble p = 0.0011). More and larger colonies formed for TUSC3 silenced tumor cells (Fig 5E and 5F). Colo357 TUSC3 shRNA2 vs Colo357 Scramble p<0.0001, Colo357 TUSC3 shRNA3 vs Colo357 Scramble p<0.0001). Then, Matrigel based assay was used to analyze the effects of TUSC3 knockdown on migration and extracellular matrix (ECM) invasion of pancreatic cancer cells. With 10% FCS as attractant, TUSC3 silenced cells displayed increased migration and invasiveness through the insert membrane in contrast to the control cells (Fig 5G, Colo357 TUSC3 shRNA2 vs Colo357 Scramble p<0.0001 Colo357 TUSC3 shRNA3 vs Colo357 Scramble p<0.0001; Fig 5H and 5I, Colo357 TUSC3 shRNA2 vs Colo357 Scramble p<0.0001, Colo357 TUSC3 shRNA3 vs Colo357 Scramble p<0.0001)). A wound healing assay further supported these results, which shows increased migration and consequent enhanced closure of the epithelial monolayer of cells in low serum conditions (5%FCS) after 18 hours (Fig 5J).

### Decreased TUSC3 promotes liver metastasis in orthotopic implanted mice model

**Fig 6:**
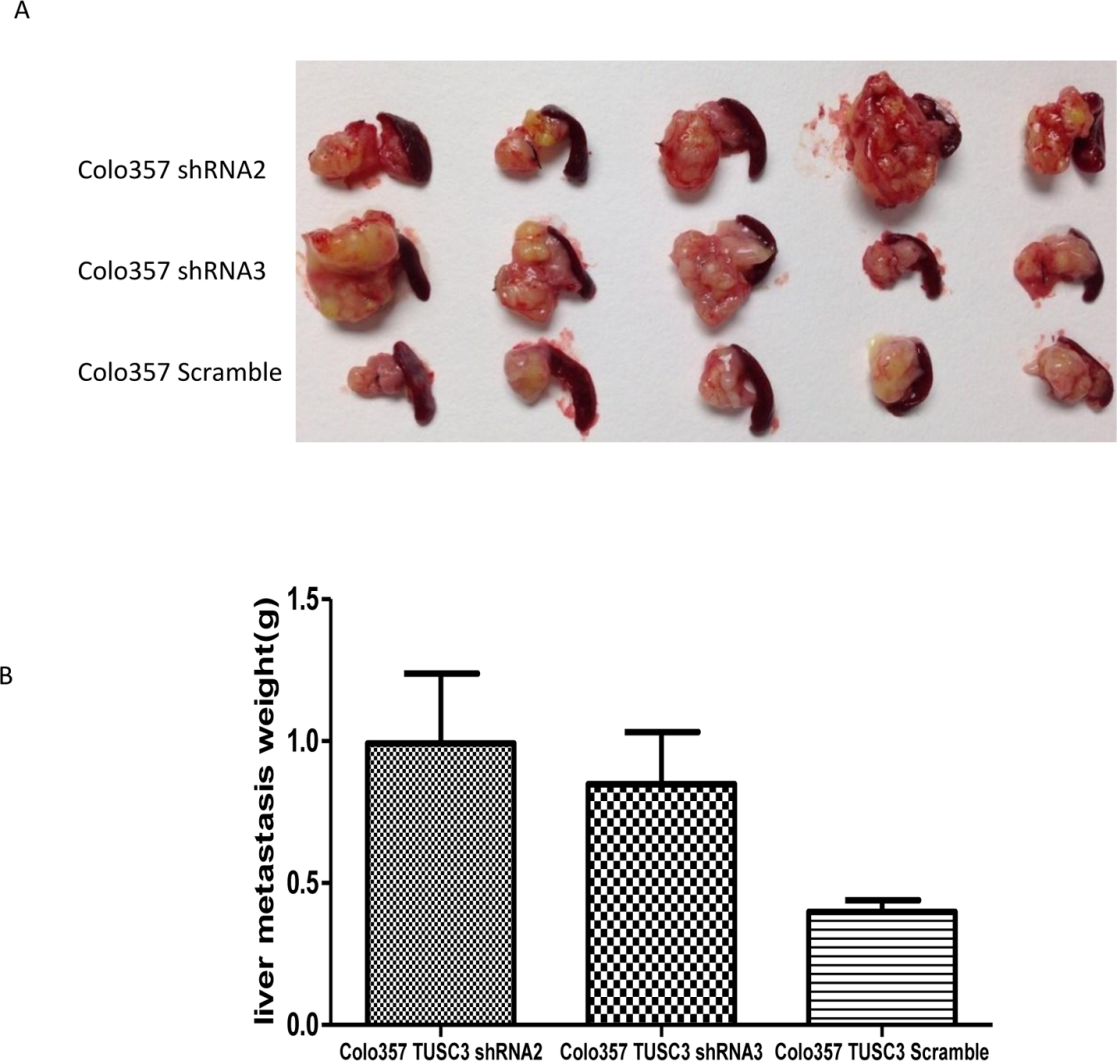
TUSC3 silenced pancreatic tumor cells exhibit more potential of liver metastasis in an orthotopic Balb/C nude mouse model. (A) Gross appearance of the resected liver specimens from mice injected with two groups of TUSC3 silenced PC (Colo357 shRNA2 and Colo357 shRNA3) and control (Colo357 Scramble). Large liver metastases are visible in group Colo357 shRNA2 and Colo357 shRNA3. (B) The liver metastases were weighted and compared using t-test.

To further explore the effect of TUSC3 on PC metastasis, we injected TUSC3 silenced tumor cells orthotopically into pancreas in nude mice and established animal models. Five weeks after injection of Colo357 shRNA2 and Colo357 shRNA3 cells, all of the mice became sick and were sacrificed. From the pictures we can apparently see the liver metastasis of TUSC3 silenced tumor is bigger than the control one (Fig 6A). Then the liver metastases were weighted and compared with t-text, Colo357 shRNA2 vs Colo357 Scramble (p = 0.0444), Colo357 shRNA3 vs Colo357 Scramble (p = 0.0443). Taken together, TUSC3 silenced cells exhibited more aggressive phenotype with more metastasis appearing in liver after the mice were sacrificed. We performed IHC as well as RT-PCR to compare the TUSC3 level between the primary foci and metastatic lesions from the orthotopic injected model samples. Compared with samples from mice injected with TUSC3 scramble cell lines, those from TUSC3 shRNA2 and TUSC3 shRNA3 show decreased expression levels of TUSC3 both with IHC (S1 Fig) and at mRNA level (S2 Fig).

## Discussion

The current study shows that TUSC3 expression is decreased in pancreatic cancer primary specimens and its down-regulation is prognostic of poor long-term outcome. *In vitro* cell line study and *in vivo* animal model provides direct evidence suggesting its role of tumor suppressor function in pancreatic cancer initiation and progression. Furthermore, the study reveals TUSC3 expression is decreased with enhanced NF-κB activity, indicative of a novel regulation model for this molecule.

Though it has been reported TUSC3 mRNA is decreased in pancreatic cancer specimens compared with the neighboring non-cancerous tissue, and recurrent loss of 8p22, which contains TUSC3 gene is found in pancreatic cancer [17, 18], no previous study has ever detected TUSC3 expression at protein level in the disease. This is in fact quite necessary given that protein is the actual executor of gene and genetic information. In this study, we not only detected TUSC3 mRNA using cell lines, but also detected its expression at protein level with immunohistochemistry in clinical samples and western blotting in cell lines. The results are consistent and confirmed that TUSC3 is dramatically depressed in pancreatic cancer, both in primary specimens and in tumor cell lines, both at mRNA level and protein level. More interestingly, TUSC3 had reverse correlation with NF-κB activity at least in some pancreatic tumor cell lines and is in consistence with the different degrees of malignancy. IκBαM-transfected AsPC-1 mu and MDA p28 mu are less malignant with attenuated NF-κB activity, and their TUSC3 expression is increased. It is noteworthy that this negative relationship is also found in another pair of cell lines, for which the daughter cell line was acquired via consecutive injections of parental cell line into nude mice rather than by transfection manipulation. All these results suggest TUSC3 expression may be regulated by NF-κB activity, at least in some tumor cell lines. Previously, TUSC3 down-regulation has been demonstrated due to homozygous and heterozygous deletion in prostate cancer, promoter hypermethylation in ovarian cancer [25]. Though it is frequently found to be deleted in pancreatic cancer [16–18], our results suggest a novel transcriptional regulation for this molecule in this cancer entity. It is possible that TUSC3 down-regulation mechanisms vary according to different tumor types. For example, early study showed TUSC3 is not hypermethylated in colorectal carcinoma [31], however hypermethylation is the major mechanism for TUSC3 down-regulation in ovarian cancer and even carries a prognostic value [25]. It is also possible that TUSC3 expression may be regulated by different means even in the same tumor type depending on different patients or cell lines. It does not rule out other possible mechanism of regulation, such as micro-RNA-mediated silencing. Activation of NF-κB and expression of its downstream target genes such as the proinflammatory cytokines tumor necrosis factor-α and IL-1 are mechanistic links between inflammation and tumorigenesis [32]. Loss of TUSC3 can destabilize the rough ER system and induce inflation of the cisternae system and alters ER stress response signaling in prostate cancer [23] and ovarian cancer [33]. Given NF-κB’s involvement in cancer [34] and TUSC3’s involvement in N-glycosylation, the possible reverse correlation between TUSC3 and NF-κB may bridge NF-κB and N-glycosylation in cancer pathogenesis, which warrants further study.

This study demonstrated that decreased TUSC3 expression is associated with higher TNM staging in pancreatic cancer and is an independent prognostic factor predictive of poor long-term outcome for resected pancreatic cancer patients. Previous work has shown decreased TUSC3 expression at mRNA level is associated with higher grade of ovarian cancer. Later on, it was discovered that TUSC3 mRNA down-regulation originates from hypermethylation of its promoter, and the methylation status has a prognostic value in ovarian cancer [25]. In prostate cancer, though there appears a trend toward poorer outcome with decreased TUSC3 mRNA expression, the difference is not statistically significant [23]. Distinct from other studies which examined mRNA expression, our study detected TUSC3 expression at protein level in primary specimens and correlated it with pancreatic cancer TNM staging and long-term outcome and confirmed that its immunohistochemical staining has a prognostic role for this disease. Compared with mRNA testing, the specimen for immunohistochemistry staining is easier to acquire and preserve, the technique is more stable and easier to apply, and can be applied in actually every hospital pathological lab.

This study has three shortcomings. First, this is a retrospective study, therefore, it cannot avoid the inherent shortcomings of any retrospective study. Second, its prognostic role is acquired from one consecutive cohort of one single center, which needs to be confirmed with another cohort. Thirdly, the current study does not explore how TUSC3 plays the tumor suppressor role in pancreatic cancer progression or the downstream signaling pathway. In Krainer’s paper on ovarian cancer, decreased TUSC3 was regarded as involved in insufficient N-glycosylation of target proteins, which may influence the normal function of these molecules [35]. However, in another paper concerning prostate cancer, it was found that decreased TUSC3 is associated with increased N-glycosylation. Although it is possible that TUSC3’s involvement in N-glycosylation varies according to caner type and N-glycosylation does play a role in cancer initiation and progression, it is also possible that its tumor suppressor role is not directly related from its involvement in N-glycosylation. After all, it does not necessarily mean that there is a causative relationship despite of its involvement in both glycosylation and cancer pathogenesis. In prostate cancer cell model, TUSC3 loss under serum deprivation promotes Akt activity [23]. How it functions in pancreatic cancer remains to be elucidated.

To sum up, the current study shows that decreased immunohistological TUSC3 staining is a factor prognostic of poor survival in pancreatic cancer patients, TUSC3 down-regulation may be connected with enhanced NF-κB activity, and decreased TUSC3 promotes pancreatic cancer cell proliferation, invasion and metastasis. Its prognostic role may help further stratification therapy for pancreatic cancer patients and warrants continued study on its regulation and downstream signaling.

## Supporting Information

S1 Fig TUSC3 silenced pancreatic tumor cells show decreased expression levels of TUSC3 with immunohistochemistry. (DOC)

S2 Fig TUSC3 silenced pancreatic tumor cells show decreased expression levels of TUSC3 at mRNA level. (DOCX)
