# Correction: Decreased TUSC3 Promotes Pancreatic Cancer Proliferation, Invasion and Metastasis

**DOI:** 10.1371/journal.pone.0151752

**Published:** 2016-03-14

**Authors:** The PLOS ONE Staff

Fig 5 appears incorrectly in the published article. Please see the complete, correct Fig 5 and its legend here. The publisher apologizes for the error.

**Fig 5:**
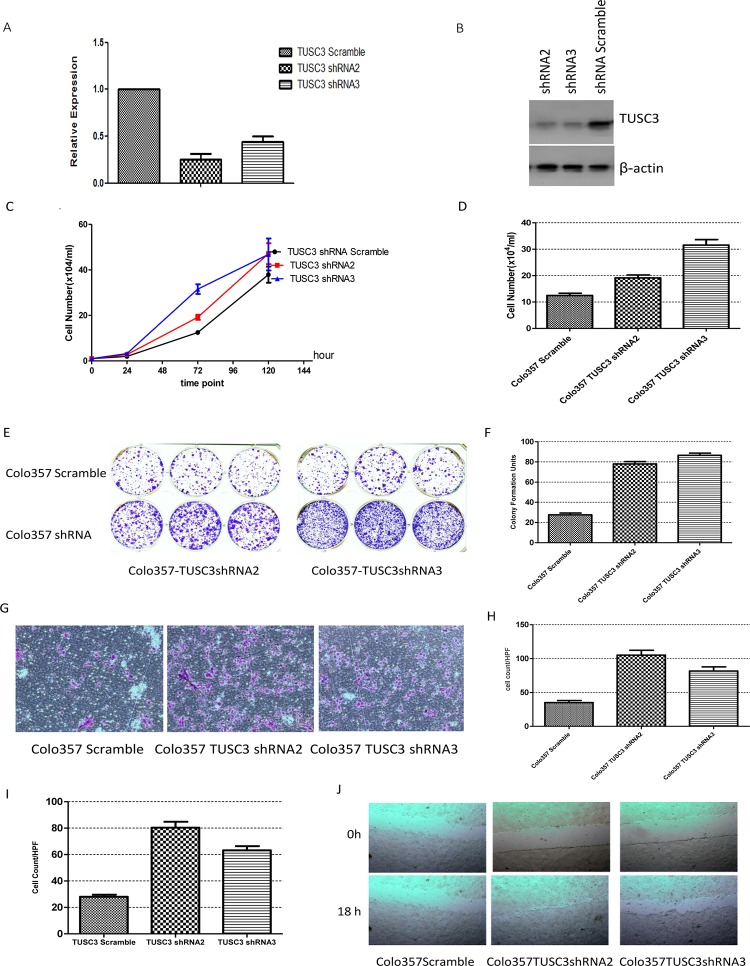
Decreased TUSC3 expression promotes tumor cell growth, migration and invasion. (A, B) TUSC3 is effectively knocked-down in Colo357 cell lines shown by (A) RT-PCR and (B) Western blot. (C, D) Proliferation is enhanced with TUSC3 knockdown, cell number counts are significantly different at 72 hours (Colo357 TUSC3 shRNA2 vs Colo357 Scramble p = 0.0086, Colo357 TUSC3 shRNA3 vs Colo357 Scramble p = 0.0011) (D). (E, F) Colony Formation is enhanced with TSUC3 knockdown (Colo357 TUSC3 shRNA2 vs Colo357 Scramble p<0.0001, Colo357 TUSC3 shRNA3 vs Colo357 Scramble p<0.0001). (G) Migration Test (Colo357 TUSC3 shRNA2 vs Colo357 Scramble p<0.0001, Colo357 TUSC3 shRNA3 vs Colo357 Scramble p<0.0001). (H, I) Invasion Test (Colo357 TUSC3 shRNA2 vs Colo357 Scramble p<0.0001, Colo357 TUSC3 shRNA3 vs Colo357 Scramble p<0.0001). (J) Wound healing test showed more rapid closure of the gap in TUSC3 silenced cells. All experiments were performed three times with representative figures shown as above.
