# Supplementary material for: Decreased TUSC3 Promotes Pancreatic Cancer Proliferation, Invasion and Metastasis
Source: PLoS One. 2016 Feb 12;11(2):e0149028. doi: 10.1371/journal.pone.0149028 (PMC4752499; doi:10.1371/journal.pone.0149028)

**S1 figure. TUSC3 silenced pancreatic tumor cells show decreased expression levels of TUSC3 with immunohistochemistry.** Immunohistochemistry of hTUSC3 of primary tumor samples from Colo357 scramble injected nude mice(A); Immunohistochemistry of hTUSC3 of primary tumor samples from Colo357 shRNA2 injected nude mice(B); Immunohistochemistry of hTUSC3 of primary tumor samples from Colo357 shRNA3 injected nude mice(C); Similar intensity of staining of liver metastasis from Colo357 Scramble cell injected tumor samples compared to primary foci (D: liver metastasis, A: primary samples from the same nude mouse).

A
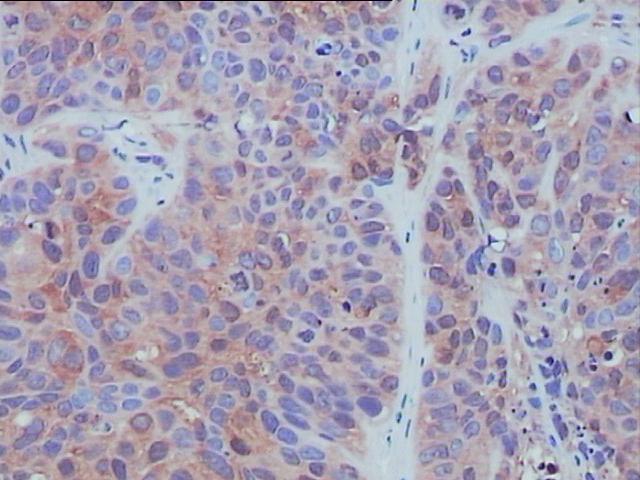
 B
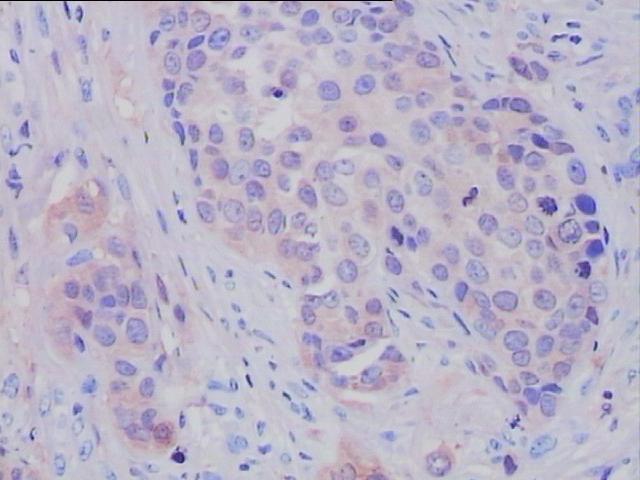


C
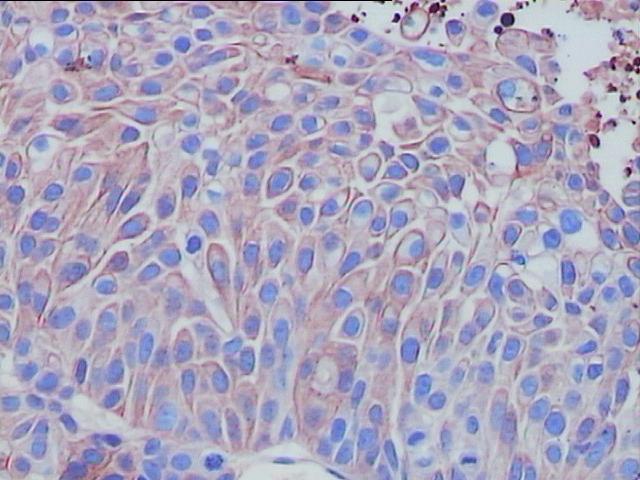
 D
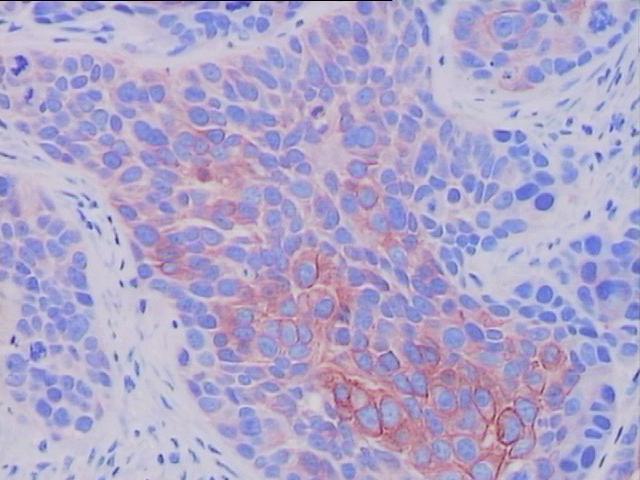

Supplement: S1 Fig — (DOC) [file pone.0149028.s001.doc]
