# Supplementary material for: Decreased TUSC3 Promotes Pancreatic Cancer Proliferation, Invasion and Metastasis
Source: PLoS One. 2016 Feb 12;11(2):e0149028. doi: 10.1371/journal.pone.0149028 (PMC4752499; doi:10.1371/journal.pone.0149028)
Supplement: S2 Fig — (DOCX) [file pone.0149028.s002.docx]

**S2 figure. TUSC3 silenced pancreatic tumor cells show decreased expression levels of TUSC3 at mRNA level.** TUSC3 mRNA levels in orthotopic transplanted tumor models samples were determined by a real-time polymerase chain reaction (PCR) assay and their levels were compared between the primary foci and liver metastasis foci. Though TUSC3 mRAN level from TUSC3 shRNA2 and shRNA3 were decreased compared with Colo357 Scramble models from primary foci(p<0.05，unpaired t-test), the expression levels are similar between the primary foci and counterpart liver metastasis foci(p> 0.05, unpaired t-test), respectively.
